# Early Trauma and Later Health: Examining the Mediation of Social Relationships in Adulthood in HRS

**DOI:** 10.1093/geroni/igab046.819

**Published:** 2021-12-17

**Authors:** Yin Liu, William Chopik, Amanda Leggett, Jooyoung Kong, Courtney Polenick

**Affiliations:** 1 Utah State University, Logan, Utah, United States; 2 Michigan State University, East Lansing, Michigan, United States; 3 University of Michigan, Ypsilanti, Michigan, United States; 4 University of Wisconsin-Madison, Madison, Wisconsin, United States; 5 University of Michigan, Ann Arbor, Michigan, United States

## Abstract

Early trauma is associated with compromised health and well-being in later life, but whether social functioning mediates the association is unclear. Participants in the Health and Retirement Study (n = 15,946) had baseline surveys in years 2006 and 2008 (T1), and were followed up twice (T2-3) every 4 years. Health outcomes included depressive symptoms, chronic health conditions, and subjective memory complaints. Social relationships were measured by contacts, relationship strains, and feelings of loneliness. Early trauma was measured by parental physical abuse and alcohol and drug problems in the family before the age of 16. Social contacts decreased over time, while relationship strains and loneliness increased especially for older adults with early trauma, which in turn mediated the associations between early trauma and poorer health in later life. The findings suggested that maintaining positive social relationships are beneficial for better health in late life, especially for individuals with early trauma exposures.

